# Associations between blood glucose level and outcomes of adult in-hospital cardiac arrest: a retrospective cohort study

**DOI:** 10.1186/s12933-016-0445-y

**Published:** 2016-08-24

**Authors:** Chih-Hung Wang, Chien-Hua Huang, Wei-Tien Chang, Min-Shan Tsai, Ping-Hsun Yu, Yen-Wen Wu, Wen-Jone Chen

**Affiliations:** 1 Department of Emergency Medicine, National Taiwan University Hospital and National Taiwan University College of Medicine, No. 7, Zhongshan S. Rd., Zhongzheng Dist., Taipei, 100 Taiwan (ROC); 2 Graduate Institute of Clinical Medicine, College of Medicine, National Taiwan University, Taipei, Taiwan; 3 Department of Emergency Medicine, Taipei Hospital, Ministry of Health and Welfare, New Taipei, Taiwan; 4 Departments of Internal Medicine and Nuclear Medicine, National Taiwan University Hospital and National Taiwan University College of Medicine, Taipei, Taiwan; 5 Department of Nuclear Medicine and Cardiology Division of Cardiovascular Medical Center, Far Eastern Memorial Hospital, New Taipei, Taiwan; 6 National Yang-Ming University School of Medicine, Taipei, Taiwan; 7 Department of Emergency Medicine, Lotung Poh-Ai Hospital, Yilan, Taiwan

**Keywords:** Heart arrest, Cardiopulmonary resuscitation, Critical care, Glucose, Diabetes mellitus, Hyperglycemia

## Abstract

**Background:**

We intended to analyse the associations between blood glucose (BG) level and clinical outcomes of in-hospital cardiac arrest (IHCA).

**Methods:**

We conducted a retrospective observational study in a single medical centre and evaluated patients who experienced IHCA between 2006 and 2014. We used multivariable logistic regression analysis to study associations between independent variables and outcomes. We calculated the mean BG level for each patient by averaging the maximum and minimum BG levels in the first 24 h after arrest, and we used mean BG level for our final analysis.

**Results:**

We included a total of 402 patients. Of these, 157 patients (39.1 %) had diabetes mellitus (DM). The average mean BG level was 209.9 mg/dL (11.7 mmol/L). For DM patients, a mean BG level between 183 and 307 mg/dL (10.2–17.1 mmol/L) was significantly associated with favourable neurological outcome (odds ratio [OR] 2.71, 95 % confidence interval [CI] 1.18–6.20; *p* value = 0.02); a mean BG level between 147 and 317 mg/dL (8.2–17.6 mmol/L) was significantly associated with survival to hospital discharge (OR 2.38, 95 % CI 1.26–4.53; p value = 0.008). For non-DM patients, a mean BG level between 143 and 268 mg/dL (7.9–14.9 mmol/L) was significantly associated with survival to hospital discharge (OR 2.93, 95 % CI 1.62–5.40; p value < 0.001).

**Conclusions:**

Mean BG level in the first 24 h after cardiac arrest was associated with neurological outcome for IHCA patients with DM. For neurological and survival outcomes, the optimal BG range may be higher for patients with DM than for patients without DM.

**Electronic supplementary material:**

The online version of this article (doi:10.1186/s12933-016-0445-y) contains supplementary material, which is available to authorized users.

## Background

In the United States, approximately 209,000 patients experience in-hospital cardiac arrest (IHCA) each year [1]. Despite ongoing efforts to improve the quality of cardiopulmonary resuscitation (CPR), outcomes following IHCA remain poor. Only 20 % of IHCA patients survive to hospital discharge, and, if patients survive, as many as 28 % suffer from significant neurological disability [2].

Dysregulated glucose homeostasis following CPR is common [3]. Studies have shown that high blood glucose (BG) levels after return of spontaneous circulation (ROSC) are associated with increased mortality and poor neurological outcome for patients who experience out-of-hospital cardiac arrest (OHCA) [4–7]. For IHCA patients, Beiser et al. reported that for patients without diabetes mellitus (DM), both hypoglycaemia and hyperglycaemia were associated with decreased survival odds; however, for patients with DM, there was little association between BG level and survival, except with extreme hyperglycaemia [8]. Associations between BG level and neurological outcome for IHCA patients have not been reported.

The American Heart Association (AHA) guidelines [9] do not recommend a target BG range for post-ROSC patients; the European Resuscitation Council (ERC) guidelines [10] suggest that the BG level be maintained below 180 mg/dL (10 mmol/L) in these patients and that hypoglycaemia should be avoided. Although the harmful effects of hypoglycaemia on survival [11] and neurological recovery [12] are well established, the BG level above which hyperglycaemia would worsen neurological outcome is unknown.

The brain is an obligate glucose consumer, so strict glucose control might restrict the supply of glucose to brain tissue and, hence, worsen brain injury. Oksanen et al. [13] conducted the only randomized controlled trial that compared strict BG control (target 72–108 mg/dL [4–6 mmol/L]) and moderate control (target 108–144 mg/dL [6–8 mmol/L]) in OHCA patients treated with therapeutic hypothermia. They reported that survival rates did not differ significantly between the two treatment groups; furthermore, they noted that levels of S-NSE, a surrogate marker for brain injury, increased in the strict BG control group [13].

In the current study, we intended to analyse the associations between BG level and clinical outcomes of IHCA, especially neurological outcome. Since the presence of DM may modify the body’s physiologic response to BG level [8, 14], we also attempted to identify the optimal BG levels for IHCA patients with and without DM.

## Methods

### Setting

We performed this retrospective cohort study at National Taiwan University Hospital (NTUH), which is a tertiary medical centre with 2600 beds, including 220 beds in intensive care units (ICUs). This study was conducted in accordance with the amended Declaration of Helsinki. Before data collection, the Research Ethics Committee of NTUH approved this study and waived the requirement for informed consent (Reference number: 201601047RINB).

According to hospital policy, a code team is activated when cardiac arrest events occur on the general wards. A code team consists of a senior resident, several junior residents, a respiratory therapist, a head nurse, and several registered ICU nurses. Each code team member is certified to provide advanced cardiac life support and is capable of offering CPR according to current resuscitation guidelines. When cardiac arrest events occur in the ICUs, a code team is not mobilized since a sufficient number of experienced staff is always present in the ICUs. In this case, resuscitation is performed by the staff of the ICU where the cardiac arrest event occurs and staff from neighbouring ICUs.

### Participants

We screened patients who suffered IHCA at NTUH between 2006 and 2014. We included patients who met the following criteria: (1) age 18 years or older, (2) documented absence of pulse with performance of chest compression for at least 2 min, (3) no documentation of a do-not-resuscitate order, and (4) achievement of sustained ROSC (i.e., ROSC ≥ 20 min without resumption of chest compression). If multiple cardiac arrest events occurred in a single patient, only the first event of the same hospitalization was recorded. We excluded patients without any measurements of BG level within the first 24 h after sustained ROSC. We also excluded patients who suffered a cardiac arrest related to major trauma.

### Data collection and outcome measures

We recorded the following information for each patient: age, gender, comorbidities (defined in Additional file 1: Table S1), variables derived from the Utstein template [15], critical interventions implemented at the time of cardiac arrest or after sustained ROSC, and the maximum and minimum BG levels measured during the 24 h after sustained ROSC. For each patient, we calculated the mean BG level by averaging the maximum and minimum BG levels. Hyperglycaemia was defined as a BG level greater than 240 mg/dL (13.3 mmol/L); hypoglycaemia was defined as a BG level less than 70 mg/dL (3.9 mmol/L) [8].

The primary outcome was favourable neurological outcome at hospital discharge, and the secondary outcome was survival to hospital discharge. Favourable neurological outcome was defined as a score of 1 or 2 on the cerebral performance category (CPC) scale [16]. The CPC scale [16] is a validated 5-point scale of neurological disability (1, good cerebral performance; 2, moderate cerebral disability; 3, severe cerebral disability; 4, coma/vegetative state; 5, death). Patients with a CPC score of 1 or 2 had sufficient cerebral function to live independently. We retrospectively determined the CPC score for each patient by reviewing medical records.

### Statistical analysis

We used R 2.15.3 software (R Foundation for Statistical Computing, Vienna, Austria) for data analysis. Categorical data are expressed as counts and proportions; continuous data are expressed as means and standard deviations. We compared categorical variables with the Fisher’s exact test, and we compared continuous variables with the Wilcoxon rank-sum test. A two-tailed *p* value of less than 0.05 was considered statistically significant.

We selected the odds ratio (OR) as the outcome measure and we performed multivariable logistic regression analyses to examine the associations between independent variables and outcomes. Among all indicators of BG control, we selected mean BG level for use in the regression analyses. We considered all available independent variables in the regression model, regardless of whether they were significant by univariate analysis. We applied the stepwise variable selection procedure (with iterations between the forward and backward steps) to obtain the final regression model. Significance levels for entry and to stay were set at 0.15 to avoid exclusion of potential candidate variables. We calculated the final regression model by excluding individual variables with a *p* value greater than 0.05 until all regression coefficients were statistically significant.

We used generalized additive models (GAMs) [17] to examine the nonlinear effects of continuous variables and, if necessary, to identify the appropriate cut-off point(s) for dichotomizing a continuous variable during the variable selection procedure. We tested the interactions between DM and mean BG level during the model-fitting process. We assessed the goodness-of-fit of the fitted regression model using *c*-statistics, adjusted generalized *R*^2^, and the Hosmer–Lemeshow goodness-of-fit test.

## Results

A total of 1537 adult patients received chest compressions for at least 2 min between 2006 and 2014. Of these, we excluded 723 patients because they did not achieve sustained ROSC, 403 patients because they did not have any measurements of BG level, and 9 patients because they experienced trauma-related cardiac arrest. We enrolled the remaining 402 patients for further analysis. The results of comparison between patients with and without post-ROSC BG level were presented in Additional file 2: Tables S2 and Additional file 3: Table S3.

Tables 1 and 2 provide the features of cardiac arrest events before, during, and after CPR for all patients in the cohort. The mean age of the patients was 65.4 years. There were 157 patients (39.1 %) with DM. A total of 138 cardiac arrest events (34.3 %) occurred in the ICUs and 217 events (54.0 %) occurred on the general wards. The majority (82.3 %) of initial rhythms were non-shockable rhythms, including pulseless electrical activity and asystole. The average CPR duration was 18.2 min. The average mean BG level was 209.9 mg/dL (11.7 mmol/L). In all, 199 patients (49.5 %) had at least one episode of hyperglycaemia and 55 patients (13.7 %) had at least one episode of hypoglycaemia. Only 130 patients (32.3 %) survived to hospital discharge; of these, 70 patients (17.4 %) demonstrated favourable neurological status. The characteristics and outcomes of patients stratified by DM are reported in Additional file 4: Tables S4 and Additional file 5: Table S5.

**Table 1:** Baseline characteristics of study patients stratified by neurological outcome

Variables	All patients (n = 402)	Patients with favourable neurological outcome at hospital discharge (n = 70)	Patients without favourable neurological outcome at hospital discharge (n = 332)	*p* value
Age, y (SD^a^)	65.4 (15.7)	62.5 (14.0)	66.1 (16.0)	0.03
Male, n (%)	243 (60.4)	52 (74.3)	191 (57.5)	0.01
Comorbidities, n (%)				
Heart failure	109 (27.1)	25 (35.7)	84 (25.3)	0.08
Myocardial infarction	60 (14.9)	19 (27.1)	41 (12.3)	0.003
Arrhythmia	91 (22.6)	17 (24.3)	74 (22.3)	0.75
Hypotension	111 (27.6)	17 (24.3)	94 (28.3)	0.56
Respiratory insufficiency	272 (67.7)	40 (57.1)	232 (69.9)	0.05
Renal insufficiency	179 (44.5)	22 (31.4)	157 (47.3)	0.02
Hepatic insufficiency	71 (17.7)	7 (10)	64 (19.3)	0.08
Metabolic or electrolyte abnormality	83 (20.6)	10 (14.3)	73 (22.0)	0.19
Diabetes mellitus	157 (39.1)	31 (44.3)	126 (38.0)	0.35
Baseline evidence of motor, cognitive, or functional deficits	177 (44.0)	23 (32.9)	154 (46.4)	0.05
Acute stroke	22 (5.5)	2 (2.9)	20 (6.0)	0.39
Favourable neurological status 24 h before cardiac arrest	215 (53.5)	50 (71.4)	165 (49.7)	<0.001
Bacteraemia	31 (7.7)	2 (2.9)	29 (8.7)	0.14
Metastatic cancer or any blood borne malignancy	65 (16.2)	1 (1.4)	64 (19.3)	<0.001

^a^SD standard deviation

**Table 2:** Features, interventions, and outcomes of cardiac arrest events stratified by neurological outcome

Variables	All patients (n = 402)	Patients with favourable neurological outcome at hospital discharge (n = 70)	Patients without favourable neurological outcome at hospital discharge (n = 332)	*p* value
Arrest at night, n (%)	240 (59.7)	41 (58.6)	199 (59.9)	0.89
Arrest on weekend, n (%)	110 (27.4)	17 (24.3)	93 (28.0)	0.56
Arrest location, n (%)				0.01
Intensive care unit	138 (34.3)	30 (42.9)	108 (32.5)	
General ward	217 (54.0)	27 (38.6)	190 (57.2)	
Others	47 (11.7)	13 (18.6)	34 (10.2)	
Witnessed arrest, n (%)	255 (63.4)	51 (72.9)	204 (61.4)	0.08
Monitored status, n (%)	231 (57.6)	49 (71.0)	182 (54.8)	0.01
Shockable rhythm, n (%)	71 (17.7)	28 (40)	43 (13.0)	<0.001
Critical care interventions in place at time of arrest, n (%)				
Mechanical ventilation	72 (17.9)	10 (14.3)	62 (18.7)	0.49
Antiarrhythmics	32 (8.0)	7 (10)	25 (7.5)	0.47
Vasopressors	128 (31.8)	24 (34.3)	104 (31.3)	0.67
Dialysis	29 (7.2)	3 (4.3)	26 (7.8)	0.45
Pulmonary artery catheter	7 (1.7)	3 (4.3)	4 (1.2)	0.10
Intra-aortic balloon pumping	6 (1.5)	2 (2.9)	4 (1.2)	0.28
CPR^a^ duration, min (SD^b^)	18.2 (16.0)	12.7 (10.2)	19.3 (16.7)	<0.001
Vital signs during the first 24 h after sustained ROSC^*c*^				
Fever, n (%)	105 (26.1)	17 (24.3)	88 (26.5)	0.77
Post-ROSC hypotension, n (%)	40 (10.0)	1 (1.4)	39 (11.7)	0.007
Indicators of glucose control during the first 24 h after sustained ROSC				
Maximum glucose level, mg/dl (SD)	259.0 (117.4)	264.6 (112.3)	257.8 (118.5)	0.67
Minimum glucose level, mg/dl (SD)	160.8 (93.6)	158.2 (60.4)	161.4 (99.2)	0.45
Mean glucose level, mg/dl (SD)	209.9 (92.7)	211.4 (75.1)	209.6.8 (96.1)	0.59
Hyperglycaemia, n (%)	199 (49.5)	36 (51.4)	163 (49.1)	0.79
Hypoglycaemia, n (%)	55 (13.7)	0 (0)	55 (16.6)	<0.001
Post-ROSC interventions, n (%)				
Extracorporeal membrane oxygenation	40 (10.0)	10 (14.3)	30 (9.0)	0.19
Therapeutic hypothermia	11 (2.7)	3 (4.3)	8 (2.4)	0.41
Percutaneous coronary intervention	32 (8.0)	18 (25.7)	14 (4.2)	<0.001
Survival for 24 h, n (%)	296 (73.6)	70 (100)	226 (68.1)	<0.001
Survival to hospital discharge, n (%)	130 (32.3)	70 (100)	60 (18.1)	<0.001

^a^ *CPR* cardiopulmonary resuscitation

^b^ *SD* standard deviation

^c^ *ROSC* return of spontaneous circulation

We placed all independent variables listed in Tables 1 and 2, as well as the pre-specified interaction terms, in the regression analysis for variable selection. The GAM plot demonstrated the non-linear association between logit (p), where p represented the probability for clinical outcomes, including favourable neurological outcome and survival to hospital discharge (Additional file 6: Figures S1–S4) and mean BG level. If logit (p) was greater than zero, the odds for favourable clinical outcomes were greater than one. Therefore, the mean BG level was transformed into a binary variable during the model-fitting process according to each identified optimal range of BG level.

Tables 3 and 4 provide the ORs of factors that significantly correlated with clinical outcomes. For patients with DM, a mean BG level between 183 and 307 mg/dL (10.2–17.1 mmol/L) was significantly associated with favourable neurological outcome (OR 2.71, 95 % confidence interval [CI] 1.18–6.20; *p* value = 0.02); a mean BG level between 141 and 317 mg/dL (8.2 and 17.6 mmol/L) was significantly associated with survival to hospital discharge (OR 2.38, 95 % CI 1.26–4.53; *p* value = 0.008). For patients without DM, the identified optimal mean BG level between 142 and 250 mg/dL (7.9–13.9 mmol/L) was not significantly associated with favourable neurological outcome (OR 1.38, 95 % CI 0.67–2.86; *p* value = 0.38), but a mean BG level between 143 and 268 mg/dL (7.9–14.9 mmol/L) was significantly associated with survival to hospital discharge (OR 2.93, 95 % CI 1.62–5.40; *p* value < 0.001).

**Table 3:** Multiple logistic regression model with favourable neurological outcome at hospital discharge as the dependent variable

Independent variable^a^	Odds ratio	95 % confidence interval	*p* value
Age between 30 and 70 years	2.32	1.21–4.59	0.01
Male	2.21	1.13–4.51	0.02
Renal insufficiency	0.45	0.23–0.86	0.02
DM * Mean glucose level between 183 and 307 *mg/dl*	*2.71*	*1.18*–*6.20*	*0.02*
Without DM * Mean glucose level between 142 and 250 *mg/dl*	*1.38*	*0.67*–*2.86*	*0.38*
Baseline evidence of motor, cognitive, or functional deficits	0.41	0.20–0.80	0.01
Favourable neurological status 24 h before cardiac arrest	3.76	1.88–7.85	<0.001
Metastatic cancer or any blood borne malignancy	0.05	0.003–0.26	0.005
Arrest on general ward	0.44	0.22–0.87	0.02
Shockable rhythm	2.84	1.37–5.88	0.005
CPR^b^ duration	0.95	0.92–0.98	0.002
Post-ROSC^c^ percutaneous coronary intervention	2.76	1.09–7.02	0.03

Goodness-of-fit assessment: n = 402, adjusted generalized *R* ^*2*^ = 0.40, the estimated area under the receiver operating characteristic curve = 0.86, and the Hosmer and Lemeshow goodness-of-fit Chi Squared test *p* = 0.79

^a^The display of independent variables is arranged in order of these variables in Tables 1 and 2

^b^ *CPR* cardiopulmonary resuscitation

^c^ *ROSC* return of spontaneous circulation

**Table 4:** Multiple logistic regression model with survival to hospital discharge as the dependent variable

Independent variable^*a*^	Odds ratio	95 % confidence interval	*p* value
Age	0.98	0.97–1.00	0.02
Hypotension	0.48	0.26–0.86	0.02
Renal insufficiency	0.59	0.35–0.98	0.04
Hepatic insufficiency	0.32	0.14–0.69	0.006
DM * Mean glucose level between 141 and 317 *mg/dl*	*2.38*	*1.26*–*4.53*	*0.008*
Without DM * Mean glucose level between 143 and 268 *mg/dl*	*2.93*	*1.62*–*5.40*	<*0.001*
Favourable neurological status 24 h before cardiac arrest	1.72	1.04–2.87	0.04
Metastatic cancer or any blood borne malignancy	0.23	0.09–0.53	<0.001
Shockable rhythm	2.70	1.48–5.00	0.001
CPR^*b*^ duration	0.96	0.94–0.98	<0.001
Post-ROSC^*c*^ hypotension	0.07	0.004–0.36	0.01

Goodness-of-fit assessment: n = 402, adjusted generalized *R* ^*2*^ = 0.36, the estimated area under the receiver operating characteristic (ROC) curve = 0.82, and the Hosmer and Lemeshow goodness-of-fit Chi Squared test *p* = 0.69

^*a*^The display of independent variables is arranged by the order of these variables in Tables 1 and 2

^*b*^ *CPR* cardiopulmonary resuscitation

^*c*^ *ROSC* return of spontaneous circulation

## Discussion

### Main findings

In this retrospective observational study, we found that post-ROSC BG level is associated with neurological and survival outcomes for IHCA patients. The optimal BG level for IHCA patients might differ according to the presence or absence of DM. Less stringent post-ROSC glycaemic control than that recommended by the ERC (i.e., maintaining the BG level below 180 mg/dL or 10 mmol/L [10] might be appropriate for some patients. Except BG level, other identified significant prognostic factors of IHCA were consistent with those reported from previous studies [18].

### Comparison with previous studies

In studies of OHCA outcomes, hyperglycaemia has been associated with poor neurological recovery [4–7]. For OHCA patients with ventricular fibrillation, Müllner et al. [4] and Nurmi et al. [5] indicated that elevated BG level in the early post-ROSC phase was associated with unfavourable neurological outcome. However, comorbidities were not considered in the statistical analyses of either of these studies [4, 5]. Kaukonen et al. [19] demonstrated that hyperglycaemia in critically ill patients may simply be an indicator of illness severity and that the association between hyperglycaemia and mortality was attenuated when lactate levels were considered in the regression analyses. Furthermore, Steingrub et al. [20] indicated that an association between the early post-ROSC BG level and neurologic outcome might not exist because a higher early post-ROSC BG level may only be an epiphenomenon of prolonged arrest duration, which itself would cause poor clinical outcomes. Without considering comorbidities and peri-CPR circumstances [4, 5], the association between hyperglycaemia and neurological outcome might be biased.

For OHCA patients receiving therapeutic hypothermia, Kim et al. [7] noted that elevated BG level in the early post-ROSC phase correlated with poor neurological recovery. However, Losert et al. [21] reported that moderate elevation of BG level at 12 h after ROSC was associated with favourable neurological outcome in non-DM patients. For OHCA patients who survived longer than 48 h after ROSC, Daviaud et al. [6] demonstrated that a high median BG level over this 48-h period was associated with poor neurological outcome. Nevertheless, the mode of death differs between OHCA and IHCA patients, with a higher proportion of patients dying from neurological disability in the former group [22]. The proportion of IHCA patients receiving therapeutic hypothermia is also low [23]. Therefore, it is unclear whether the results of these OHCA studies [6, 7, 21] can be applied to IHCA patients.

Beiser et al. [8] were the first to explore the association between BG level and survival outcome for IHCA by analysing a representative nationwide database. They demonstrated that the patterns of association between BG level and survival outcome were different for DM and non-DM patients. Still, in the study by Beiser et al. [8], maximum and minimum BG levels over the first 24 h after ROSC were analysed separately in the regression analysis, making the results difficult to interpret and apply. Even if BG levels were the same, the survival odds differed depending on the category (i.e. minimum or maximum BG level) to which the value was ascribed, which could only be known retrospectively [8].

### Interpretation of current analysis

We chose to use the mean BG level in our analysis to avoid the issue raised by Beiser et al. [8] Our findings indicate that the patterns of association between BG level and survival were similar to those reported by Beiser et al. [8], demonstrating reverse-U shaped curves (Additional file 6: Figures S3, S4) with lower survival odds for patients with hypoglycaemia or hyperglycaemia. Furthermore, for IHCA patients with DM, a BG level between 183 and 307 mg/dL (10.2–17.1 mmol/L) was significantly associated with favourable neurological outcome; for IHCA patients without DM, the association was not significant, which may be due to inadequate statistical power. Finally, our study demonstrated that the upper bounds of optimal BG levels for both neurological and survival outcomes were higher in DM patients than in non-DM patients. This result corresponds to the findings of Egi et al. [14], who reported that critically ill DM patients were more capable of tolerating the influences of hyperglycaemia than patients without DM, and DM patients experienced significantly lower mortality at all levels of hyperglycaemia. Nevertheless, for both DM and non-DM patients, the upper limits of the optimal BG range were higher than the recommendations of the ERC [10].

The harm caused by hypoglycaemia in critically ill patients is well recognized [11, 12], but the influence of hyperglycaemia is more controversial. Because of the ease of application in statistical analysis and clinical practice, we selected mean BG level for our analysis. Mean values are subject to influences of extreme values and may, therefore, contribute to the elevated optimal BG level ranges. In previous studies, mean [24], median [6], and maximum/minimum [8] BG levels over certain periods have been used in analyses. Whether a single value of BG could be used to assess glucose control over time is unknown. Eslami et al. [25] documented 30 different published methods to describe the quality of glucose control, and the various indicators of glucose control adopted in different studies [4–8, 20, 21, 24] might be the reason why the AHA [9] does not recommend a specific target BG level for post-ROSC patients. Future study is warranted to establish the optimal indicator to depict and analyse the dynamics and impacts of BG levels.

### Protective role of moderate hyperglycaemia

Despite the limitation of using a single BG value to represent the dynamics of BG levels, the observed role of moderate hyperglycaemia in our study merits further discussion. Sustained hyperglycaemia could worsen secondary brain injury and lead to poor neurological outcome [26, 27]. However, the definition of hyperglycaemia in terms of BG level has not been well established as it relates to brain injury. The human brain depends almost solely on the availability of systemic glucose supply to maintain normal metabolism. After ischemic insults, the transport of glucose to brain tissues may become inadequate to satisfy cerebral metabolism [28]. When cerebral perfusion is compromised, moderate hyperglycaemia may facilitate glucose transport through the elevated BG diffusion gradient that maximizes cellular glucose uptake [29]. Studies have shown that normalization of BG levels in critically ill patients with brain injury may be associated with greater risk of critical reductions in brain glucose levels and energy crises [30, 31]. Therefore, acute stress hyperglycaemia noted during the early post-ROSC phase might be a physiologic, rather than a pathologic, response and attempts at interfering with this complex adaptive response may be harmful rather than protective [32].

Oksanen et al. [13] indicated that, for OHCA patients, a strict BG control strategy would lead to more severe brain injuries. In other critically ill patients, strict BG control has not been shown to reduce mortality, which was probably caused by an increased incidence of hypoglycaemia [33]. On the basis of our results, relaxing the upper limit of BG levels recommended by the ERC may be justified [10]. A randomized clinical trial targeting two or more BG levels for post-arrest patients would be needed to verify this assumption. Also, as recent studies [34–36] indicated, the outcomes of DM patients following cardiac arrest might be distinct from those of non-DM patients. Future studies should address the implication of DM in the pathogenesis of mortality and morbidity after cardiac arrest.

### Study limitations

This study has several limitations that must be considered. First, this was an observational study, which can only establish an association, rather than a causal relationship, between independent and dependent variables. Second, the effects of unmeasured confounders might bias the results. Third, our study did not involve any specific protocol for BG control. We did not suggest that clinicians actively administer intravenous glucose to maintain the BG level at a certain range or that clinicians should allow permissive hyperglycaemia. Administration of intravenous glucose might be associated with worse neurological outcome for post-ROSC patients [37]. The optimal strategy to maintain BG within a certain range should be examined in a randomized controlled trial. Fourth, about half of patients who achieved sustained ROSC were excluded from our analysis because of lack of measurements of BG level after sustained ROSC. As shown in the Additional file 2: Tables S2 and Additional file 3: Table S3, although there were no significant baseline differences between patients with and without measurements of BG levels, the outcomes of the former were better than the latter. Therefore, patients without any measurement of BG levels may have more unstable haemodynamics, leading to the absence of any BG levels measured after sustained ROSC. This kind of selection bias may only be resolved through a prospective study with a protocol for monitoring post-ROSC BG levels. Finally, this was a single-centre study, which might limit the generalizability of the study conclusion. Since the guideline-based care varied significantly across hospitals [38], a multi-centre study would be needed to confirm our observation.

## Conclusions

The mean BG level over the first 24 h after ROSC is associated with neurological outcomes of IHCA patients with DM. For neurological and survival outcomes, the optimal BG range may be higher for patients with DM than for patients without DM.

## Additional files

10.1186/s12933-016-0445-y Definitions of comorbidities used in the multivariable models.
10.1186/s12933-016-0445-y Baseline characteristics of study patients stratified by the presence of measurement of blood glucose level after sustained return of spontaneous circulation.
10.1186/s12933-016-0445-y Features, interventions, and outcomes of cardiac arrest events stratified by the presence of measurement of blood glucose level after sustained return of spontaneous circulation.
10.1186/s12933-016-0445-y Baseline characteristics of study patients stratified by the presence of diabetes mellitus.
10.1186/s12933-016-0445-y Features, interventions, and outcomes of cardiac arrest events stratified by the presence of diabetes mellitus.
10.1186/s12933-016-0445-y Additional Figures.
10.1186/s12933-016-0445-y

## Abbreviations

IHCA: in-hospital cardiac arrest
CPR: cardiopulmonary resuscitation
BG: blood glucose
ROSC: return of spontaneous circulation
OHCA: out-of-hospital cardiac arrest
DM: diabetes mellitus
AHA: American Heart Association
ERC: European Resuscitation Council
NTUH: National Taiwan University Hospital
ICU: intensive care units
CPC: cerebral performance category
OR: odds ratio
GAM: generalized additive models
