# Supplementary material for: Associations between blood glucose level and outcomes of adult in-hospital cardiac arrest: a retrospective cohort study
Source: Cardiovasc Diabetol. 2016 Aug 24;15(1):118. doi: 10.1186/s12933-016-0445-y (PMC4997657; doi:10.1186/s12933-016-0445-y)
Supplement: Supplementary file 1 — 10.1186/s12933-016-0445-y Definitions of comorbidities used in the multivariable models. [file 12933_2016_445_MOESM1_ESM.docx]

Supplemental Table 1. Definitions of comorbidities used in the multivariable models

| Variables | Definitions |
| --- | --- |
| Heart failure | Documented diagnosis of congestive heart failure during this or previous admissions |
| Myocardial infarction | Documented diagnosis of myocardial ischaemia (acute coronary syndrome) or myocardial infarction during this or previous admissions |
| Arrhythmia | Documented diagnosis of a cardiac arrhythmia |
| Hypotension | Evidence of hypotension within 4 h before the event, defined by ANY of the following:  1. SBP < 90 or MAP < 60 mmHg  2. vasopressor or inotropic requirement after volume expansion (except for dopamine ≤ 3 mcg/kg/min)  3. Intra-aortic balloon pump |
| Respiratory insufficiency | Evidence of acute or chronic respiratory insufficiency within 4 h before the event, defined by ANY of the following:  1. PaO2/FiO2 ratio < 300 (in the absence of pre-existing documented cyanotic heart disease)  2. PaO2 < 60 mm Hg (in the absence of pre-existing documented cyanotic heart disease)  3. SaO2 < 90%, (in the absence of pre-existing documented cyanotic heart disease)  4. PaCO2, EtCO2 or TcCO2 > 50 mmHg  5. Spontaneous respiratory rate > 40/min or < 5/min  6. Need for non-invasive ventilation (e.g., Bag-Valve-Mask, Mask CPAP or BiPAP, Nasal CPAP or BiPAP, negative pressure ventilation)  7. Need for ventilation via invasive airway (e.g., T-piece, assist control, IMV, pressure support, high frequency) |
| Renal insufficiency | Evidence of renal insufficiency prior to the event, defined by ANY of the following:  1. Requiring ongoing dialysis or extracorporeal filtration therapies  2. Creatinine > 2 mg/dL within 24 h before the event |
| Hepatic insufficiency | Evidence of hepatic insufficiency within 24 h before the event, defined by ANY of the following:  1. Total bilirubin > 2 mg/dL and AST > 2x normal  2. Cirrhosis |
| Metabolic or electrolyte abnormality | Evidence of metabolic/electrolyte abnormality within 4 h before the event, defined by ANY of the following:  1. Sodium < 125 or > 150 mEq/L  2. Potassium < 2.5 or > 6 mEq/L  3. Arterial pH < 7.3 or > 7.5  4. Lactate > 2.5 mmol/L  5. Blood glucose < 60 mg/dL |
| Diabetes mellitus | Documented diagnosis of type 1 or type 2 diabetes mellitus |
| Baseline evidence of motor, cognitive, or functional deficits | Evidence of a motor, cognitive, or functional baseline deficit (at time of system entry) |
| Acute stroke | Documented diagnosis of an intracranial or intraventricular haemorrhage or thrombosis during this admission |
| Favourable neurological status 24 h before cardiac arrest | Best CPC score within 24 h before cardiac arrest  CPC1: Good cerebral performance: conscious, alert, able to work, might have mild neurologic or psychologic deficit  CPC2: Moderate cerebral disability: conscious, sufficient cerebral function for independent activities of daily life; able to work in sheltered environment  CPC3: Severe cerebral disability: conscious, dependent on others for daily support because of impaired brain function; ranges from ambulatory state to severe dementia or paralysis  CPC4: Coma or vegetative state: any degree of coma without the presence of all brain death criteria; unawareness, even if appears awake (vegetative state) without interaction with environment; may have spontaneous eye opening and sleep/awake cycles  CPC5: Brain death: apnoea, areflexia, EEG silence |
| Bacteraemia | Bloodstream infection for which antibiotics have not yet been started or the infection is still being treated with antibiotics |
| Metastatic cancer or any blood borne malignancy | Any solid tissue malignancy with evidence of metastasis, or any blood borne malignancy |

BiPAP, bilevel positive airway pressure; CPAP, continuous positive airway pressure; CPC, cerebral performance category; EEG, electroencephalogram; IMV, intermittent mandatory ventilation
