# Supplementary material for: Associations between blood glucose level and outcomes of adult in-hospital cardiac arrest: a retrospective cohort study
Source: Cardiovasc Diabetol. 2016 Aug 24;15(1):118. doi: 10.1186/s12933-016-0445-y (PMC4997657; doi:10.1186/s12933-016-0445-y)
Supplement: Supplementary file 2 — 10.1186/s12933-016-0445-y Baseline characteristics of study patients stratified by the presence of measurement of blood glucose level after sustained return of spontaneous circulation. [file 12933_2016_445_MOESM2_ESM.docx]

Supplemental Table 2. Baseline characteristics of study patients stratified by the presence of measurement of blood glucose level after sustained return of spontaneous circulation

| Variables | Patients with measurement of post-ROSC*^b^* blood glucose level  (n = 402) | Patients without measurement of post- ROSC blood glucose level (n = 403) | *p*-value |
| --- | --- | --- | --- |
| Age, y (SD*^a^*) | 65.4 (15.7) | 65.4 (16.4) | 0.83 |
| Male, n (%) | 243 (60.4) | 233 (57.8) | 0.47 |
| Comorbidities, n (%) |  |  |  |
| Heart failure | 109 (27.1) | 100 (24.8) | 0.47 |
| Myocardial infarction | 60 (14.9) | 65 (16.1) | 0.70 |
| Arrhythmia | 91 (22.6) | 70 (17.4) | 0.06 |
| Hypotension | 111 (27.6) | 77 (19.1) | 0.005 |
| Respiratory insufficiency | 272 (67.7) | 299 (74.2) | 0.04 |
| Renal insufficiency | 179 (44.5) | 180 (44.7) | 1 |
| Hepatic insufficiency | 71 (17.7) | 80 (19.9) | 0.47 |
| Metabolic or electrolyte  abnormality | 83 (20.6) | 66 (16.4) | 0.12 |
| Diabetes mellitus | 157 (39.1) | 138 (34.2) | 0.16 |
| Baseline evidence of motor, cognitive, or functional deficits | 177 (44.0) | 78 (19.4) | <0.001 |
| Acute stroke | 22 (5.5) | 15 (3.7) | 0.24 |
| Favourable neurological status 24 h before cardiac arrest | 215 (53.5) | 153 (38.0) | <0.001 |
| Bacteraemia | 31 (7.7) | 46 (11.4) | 0.09 |
| Metastatic cancer or any blood borne malignancy | 65 (16.2) | 102 (25.3) | 0.002 |

*^a^*SD, standard deviation.

*^b^*ROSC, return of spontaneous circulation
