# Supplementary material for: Associations between blood glucose level and outcomes of adult in-hospital cardiac arrest: a retrospective cohort study
Source: Cardiovasc Diabetol. 2016 Aug 24;15(1):118. doi: 10.1186/s12933-016-0445-y (PMC4997657; doi:10.1186/s12933-016-0445-y)
Supplement: Supplementary file 3 — 10.1186/s12933-016-0445-y Features, interventions, and outcomes of cardiac arrest events stratified by the presence of measurement of blood glucose level after sustained return of spontaneous circulation. [file 12933_2016_445_MOESM3_ESM.docx]

Supplemental Table 3. Features, interventions, and outcomes of cardiac arrest events stratified by the presence of measurement of blood glucose level after sustained return of spontaneous circulation

| Variables | Patients with measurement of post-ROSC blood glucose level  (n = 402) | Patients without measurement of post-ROSC blood glucose level (n = 403) | *p*-value |
| --- | --- | --- | --- |
| Arrest at night, n (%) | 240 (59.7) | 240 (59.6) | 1 |
| Arrest on weekend, n (%) | 110 (27.4) | 121 (30.0) | 0.44 |
| Arrest location, n (%) |  |  | <0.001 |
| Intensive care unit | 138 (34.3) | 196 (48.6) |  |
| General ward | 217 (54.0) | 180 (44.7) |  |
| Others | 47 (11.7) | 27 (6.7) |  |
| Witnessed arrest, n (%) | 255 (63.4) | 279 (69.2) | 0.09 |
| Monitored status, n (%) | 231 (57.6) | 243 (60.2) | 0.47 |
| Shockable rhythm, n (%) | 71 (17.7) | 63 (15.6) | 0.45 |
| Critical care interventions in place at time of arrest, n (%) |  |  |  |
| Mechanical ventilation | 72 (17.9) | 75 (18.6) | 0.86 |
| Antiarrhythmics | 32 (8.0) | 36 (8.9) | 0.70 |
| Vasopressors | 128 (31.8) | 168 (41.7) | 0.004 |
| Dialysis | 29 (7.2) | 33 (8.2) | 0.69 |
| Pulmonary artery catheter | 7 (1.7) | 2 (0.5) | 0.11 |
| Intra-aortic balloon pumping | 6 (1.5) | 6 (1.5) | 1 |
| CPR*^a^* duration, min (SD*^b^*) | 18.2 (16.0) | 20.3 (20.6) | <0.001 |
| Vital signs during the first 24 h after sustained ROSC*^c^* |  |  |  |
| Fever, n (%) | 105 (26.1) | 98 (24.3) | 0.57 |
| Post-ROSC hypotension, n (%) | 40 (10.0) | 56 (13.9) | 0.10 |
| Indicators of glucose control during the first 24 h after sustained ROSC |  |  |  |
| Maximum glucose level, mg/dl (SD) | 259.0 (117.4) | NA | NA |
| Minimum glucose level, mg/dl (SD) | 160.8 (93.6) | NA | NA |
| Mean glucose level, mg/dl (SD) | 209.9 (92.7) | NA | NA |
| Hyperglycaemia, n (%) | 199 (49.5) | NA | NA |
| Hypoglycaemia, n (%) | 55 (13.7) | NA | NA |
| Post-ROSC^c^ interventions, n (%) |  |  |  |
| Extracorporeal membrane oxygenation | 40 (10.0) | 14 (3.5) | <0.001 |
| Therapeutic hypothermia | 11 (2.7) | 0 (0) | <0.001 |
| Percutaneous coronary intervention | 32 (8.0) | 17 (4.2) | 0.03 |
| Survival for 24 h, n (%) | 296 (73.6) | 205 (50.9) | <0.001 |
| Survival to hospital discharge, n (%) | 130 (32.3) | 78 (19.4) | <0.001 |
| Favourable neurological outcome at hospital discharge, n (%) | 70 (17.4) | 37 (9.2) | <0.001 |

*^a^*CPR, cardiopulmonary resuscitation

*^b^*SD, standard deviation

*^c^*ROSC, return of spontaneous circulation
