# Supplementary material for: Associations between blood glucose level and outcomes of adult in-hospital cardiac arrest: a retrospective cohort study
Source: Cardiovasc Diabetol. 2016 Aug 24;15(1):118. doi: 10.1186/s12933-016-0445-y (PMC4997657; doi:10.1186/s12933-016-0445-y)
Supplement: Supplementary file 4 — 10.1186/s12933-016-0445-y Baseline characteristics of study patients stratified by the presence of diabetes mellitus. [file 12933_2016_445_MOESM4_ESM.docx]

Supplemental Table 4. Baseline characteristics of study patients stratified by the presence of diabetes mellitus

| Variables | All patients  (n = 402) | Patients with diabetes mellitus (n = 157) | Patients without diabetes mellitus (n = 245) | *p*-value |
| --- | --- | --- | --- | --- |
| Age, y (SD*^a^*) | 65.4 (15.7) | 68.3 (11.9) | 63.6 (17.5) | 0.04 |
| Male, n (%) | 243 (60.4) | 96 (59.5) | 147 (60) | 0.84 |
| Comorbidities, n (%) |  |  |  |  |
| Heart failure | 109 (27.1) | 51 (32.5) | 58 (23.7) | 0.07 |
| Myocardial infarction | 60 (14.9) | 34 (21.7) | 26 (10.6) | 0.004 |
| Arrhythmia | 91 (22.6) | 40 (25.5) | 51 (20.8) | 0.33 |
| Hypotension | 111 (27.6) | 44 (28.0) | 67 (27.3) | 0.91 |
| Respiratory insufficiency | 272 (67.7) | 104 (66.2) | 168 (68.6) | 0.66 |
| Renal insufficiency | 179 (44.5) | 87 (55.4) | 92 (37.6) | <0.001 |
| Hepatic insufficiency | 71 (17.7) | 23 (14.6) | 48 (19.6) | 0.23 |
| Metabolic or electrolyte  abnormality | 83 (20.6) | 29 (18.5) | 54 (22.0) | 0.45 |
| Baseline evidence of motor, cognitive, or functional deficits | 177 (44.0) | 82 (52.2) | 95 (38.8) | 0.01 |
| Acute stroke | 22 (5.5) | 11 (7.0) | 11 (4.4) | 0.37 |
| Favourable neurological status 24 h before cardiac arrest | 215 (53.5) | 78 (49.7) | 137 (55.9) | 0.26 |
| Bacteraemia | 31 (7.7) | 13 (8.2) | 18 (7.3) | 0.85 |
| Metastatic cancer or any blood borne malignancy | 65 (16.2) | 15 (9.6) | 50 (20.4) | 0.004 |

*^a^*SD, standard deviation.
