# Supplementary material for: Associations between blood glucose level and outcomes of adult in-hospital cardiac arrest: a retrospective cohort study
Source: Cardiovasc Diabetol. 2016 Aug 24;15(1):118. doi: 10.1186/s12933-016-0445-y (PMC4997657; doi:10.1186/s12933-016-0445-y)
Supplement: Supplementary file 5 — 10.1186/s12933-016-0445-y Features, interventions, and outcomes of cardiac arrest events stratified by the presence of diabetes mellitus. [file 12933_2016_445_MOESM5_ESM.docx]

Supplemental Table 5. Features, interventions, and outcomes of cardiac arrest events stratified by the presence of diabetes mellitus

| Variables | All patients  (n = 402) | Patients with diabetes mellitus (n = 157) | Patients without diabetes mellitus (n = 245) | *p*-value |
| --- | --- | --- | --- | --- |
| Arrest at night, n (%) | 240 (59.7) | 90 (57.3) | 150 (61.2) | 0.47 |
| Arrest on weekend, n (%) | 110 (27.4) | 37 (23.6) | 73 (29.8) | 0.21 |
| Arrest location, n (%) |  |  |  | 0.48 |
| Intensive care unit | 138 (34.3) | 49 (31.2) | 89 (36.3) |  |
| General ward | 217 (54.0) | 87 (55.4) | 130 (53.1) |  |
| Others | 47 (11.7) | 21 (13.4) | 26 (10.6) |  |
| Witnessed arrest, n (%) | 255 (63.4) | 88 (56.1) | 167 (68.2) | 0.01 |
| Monitored status, n (%) | 231 (57.6) | 88 (56.1) | 143 (58.6) | 0.61 |
| Shockable rhythm, n (%) | 71 (17.7) | 35 (22.3) | 36 (14.7) | 0.06 |
| Critical care interventions in place at time of arrest, n (%) |  |  |  |  |
| Mechanical ventilation | 72 (17.9) | 25 (15.9) | 47 (19.2) | 0.43 |
| Antiarrhythmics | 32 (8.0) | 13 (8.3) | 19 (7.8) | 0.85 |
| Vasopressors | 128 (31.8) | 44 (28.0) | 84 (34.3) | 0.23 |
| Dialysis | 29 (7.2) | 14 (8.9) | 15 (6.1) | 0.33 |
| Pulmonary artery catheter | 7 (1.7) | 3 (1.9) | 4 (1.6) | 1 |
| Intra-aortic balloon pumping | 6 (1.5) | 3 (1.9) | 3 (1.2) | 0.68 |
| CPR*^a^* duration, min (SD*^b^*) | 18.2 (16.0) | 18.6 (14.7) | 17.9 (16.8) | 0.25 |
| Vital signs during the first 24 h after sustained ROSC*^c^* |  |  |  |  |
| Fever, n (%) | 105 (26.1) | 43 (27.4) | 62 (25.3) | 0.64 |
| Post-ROSC hypotension, n (%) | 40 (10.0) | 10 (6.4) | 30 (12.2) | 0.06 |
| Indicators of glucose control during the first 24 h after sustained ROSC |  |  |  |  |
| Maximum glucose level, mg/dl (SD) | 259.0 (117.4) | 302.1 (113.0) | 231.3 (111.8) | <0.001 |
| Minimum glucose level, mg/dl (SD) | 160.8 (93.6) | 180.6 (99.6) | 148.2 (87.4) | 0.001 |
| Mean glucose level, mg/dl (SD) | 209.9 (92.7) | 241.3 (94.9) | 189.8 (85.6) | <0.001 |
| Hyperglycaemia, n (%) | 199 (49.5) | 107 (68.2) | 92 (37.6) | <0.001 |
| Hypoglycaemia, n (%) | 55 (13.7) | 19 (12.1) | 36 (14.7) | 0.55 |
| Post-ROSC^c^ interventions, n (%) |  |  |  |  |
| Extracorporeal membrane oxygenation | 40 (10.0) | 18 (11.5) | 22 (9.0) | 0.49 |
| Therapeutic hypothermia | 11 (2.7) | 6 (3.8) | 5 (2.0) | 0.35 |
| Percutaneous coronary intervention | 32 (8.0) | 19 (12.1) | 13 (5.3) | 0.02 |
| Survival for 24 h, n (%) | 296 (73.6) | 119 (75.8) | 177 (72.2) | 0.49 |
| Survival to hospital discharge, n (%) | 130 (32.3) | 52 (33.1) | 78 (31.8) | 0.83 |
| Favourable neurological outcome at hospital discharge, n (%) | 70 (17.4) | 31 (19.7) | 39 (15.9) | 0.35 |

*^a^*CPR, cardiopulmonary resuscitation

*^b^*SD, standard deviation

*^c^*ROSC, return of spontaneous circulation
