# Supplementary material for: Associations between blood glucose level and outcomes of adult in-hospital cardiac arrest: a retrospective cohort study
Source: Cardiovasc Diabetol. 2016 Aug 24;15(1):118. doi: 10.1186/s12933-016-0445-y (PMC4997657; doi:10.1186/s12933-016-0445-y)
Supplement: Supplementary file 6 — 10.1186/s12933-016-0445-y Additional Figures. [file 12933_2016_445_MOESM6_ESM.docx]

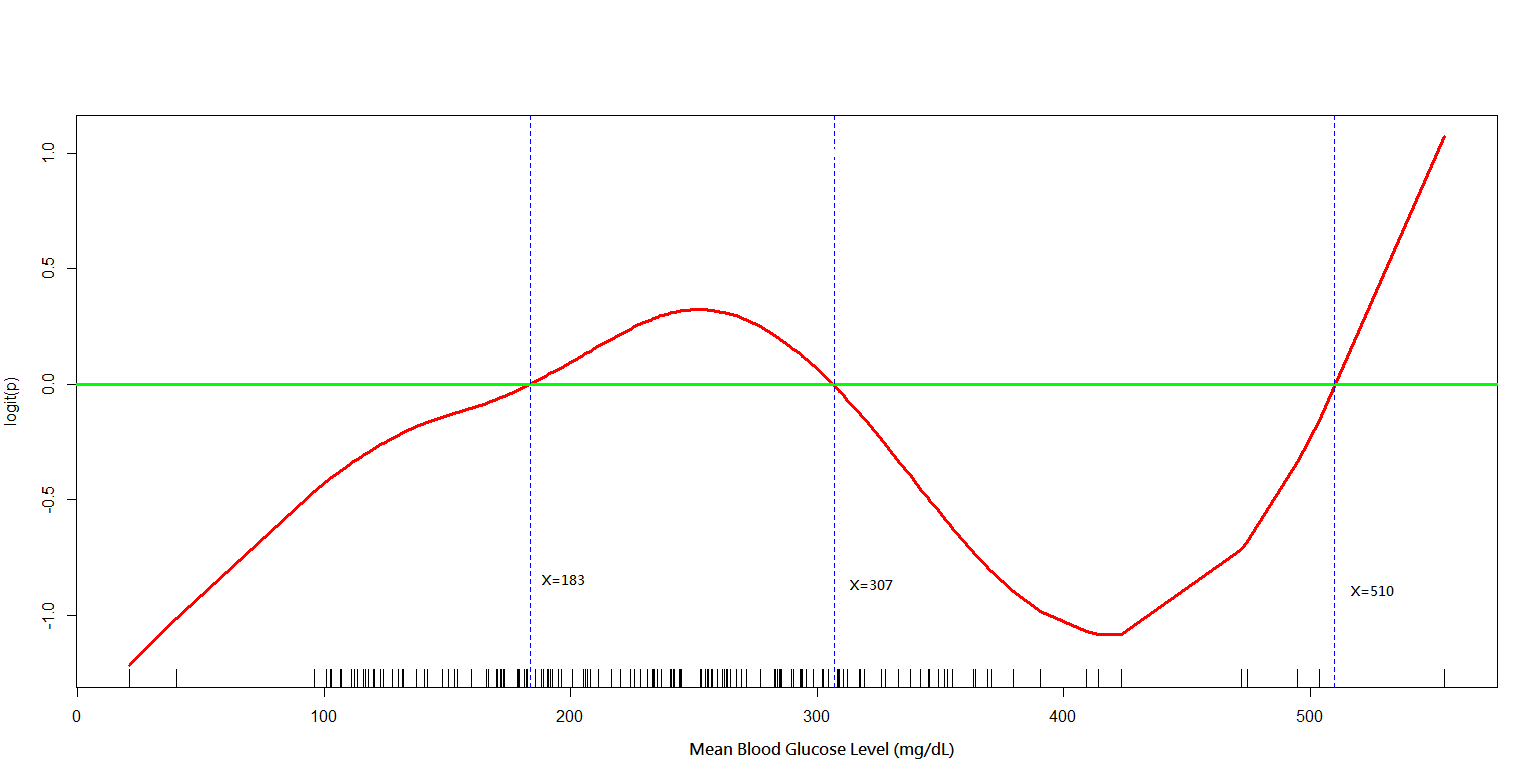


**Figure S1** Generalized additive model plot for nonparametric modelling of the effect of mean blood glucose level on the logit of probability for favourable neurological outcome at hospital discharge in patients with diabetes mellitus


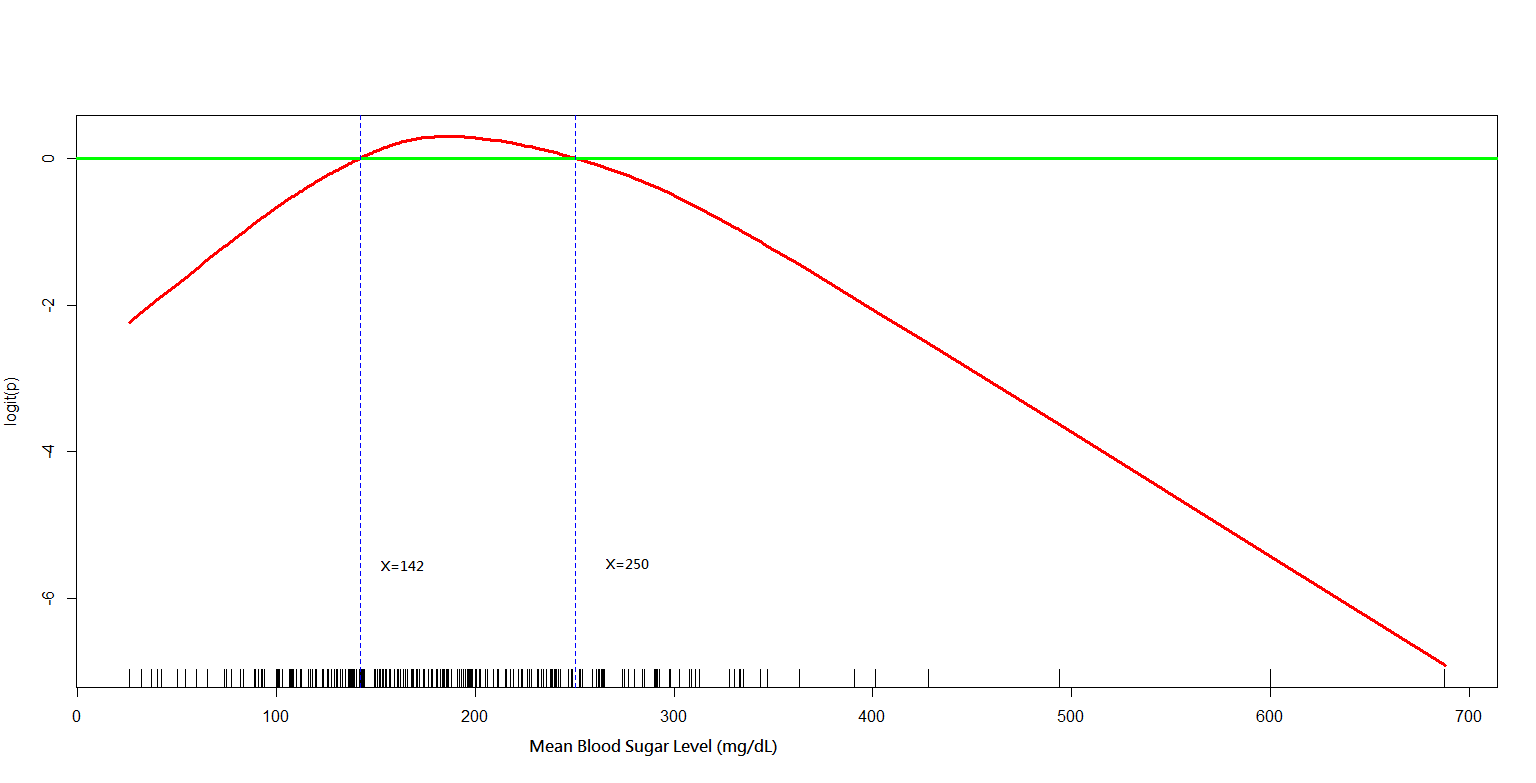


**Figure S2** Generalized additive model plot for nonparametric modelling of the effect of mean blood glucose level on the logit of probability for favourable neurological outcome at hospital discharge in patients without diabetes mellitus


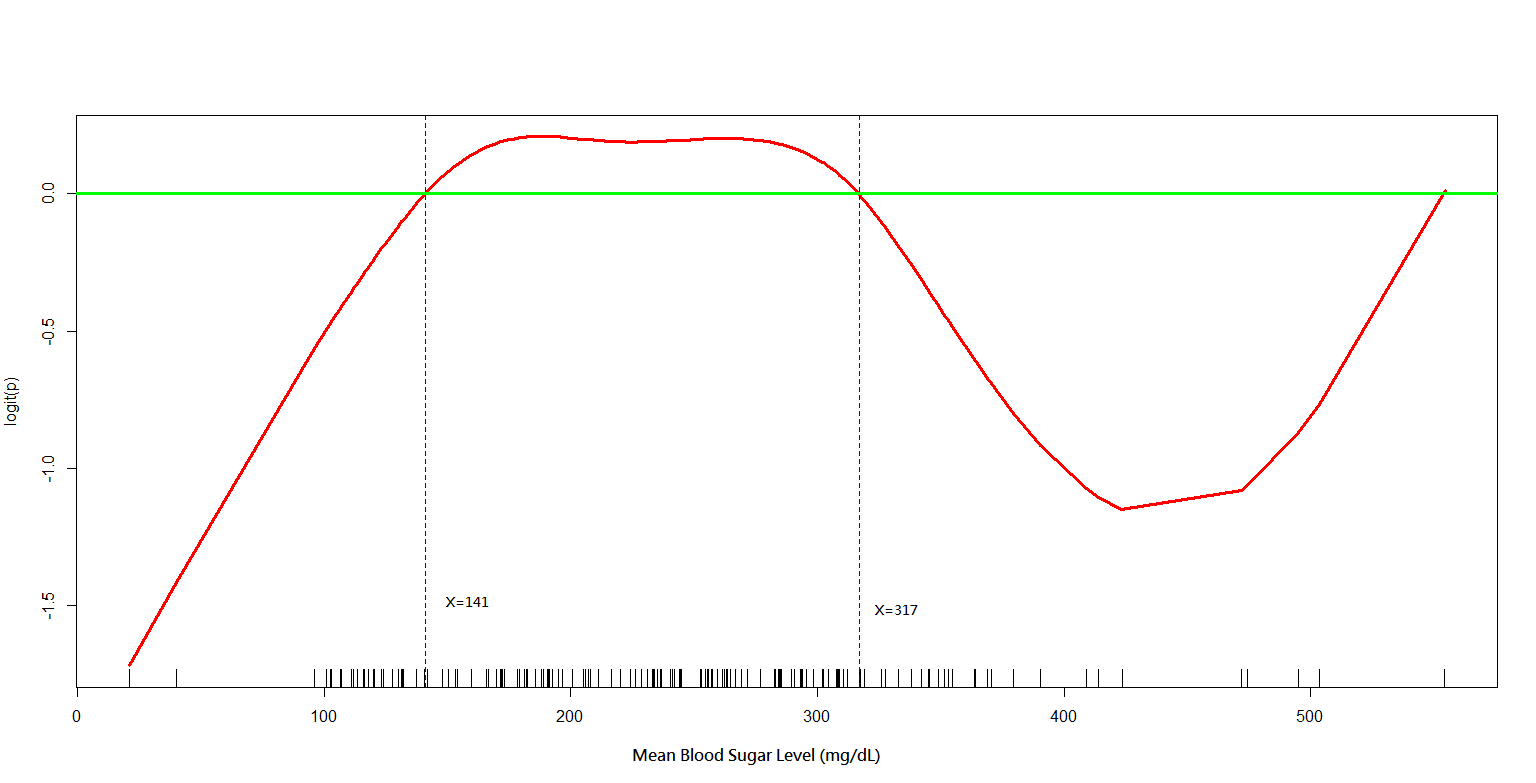


**Figure S3** Generalized additive model plot for nonparametric modelling of the effect of mean blood glucose level on the logit of probability for survival to hospital discharge in patients with diabetes mellitus


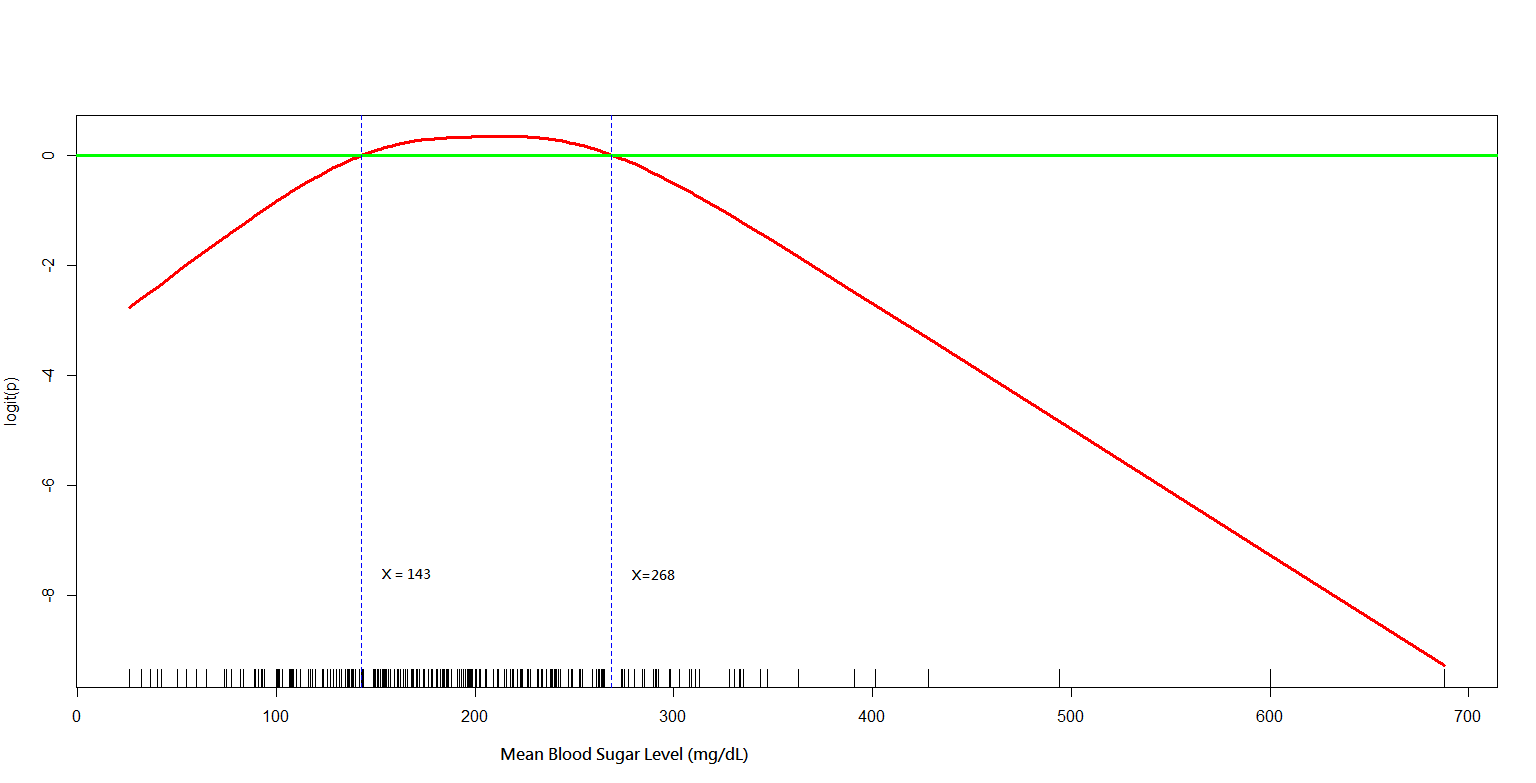


**Figure S4** Generalized additive model plot for nonparametric modelling of the effect of mean blood glucose level on the logit of probability for survival to hospital in patients without diabetes mellitus
